# Supplementary material for: Systematic assessment of gene co-regulation within chromatin domains determines differentially active domains across human cancers
Source: Genome Biol. 2021 Aug 3;22:218. doi: 10.1186/s13059-021-02436-6 (PMC8330107; doi:10.1186/s13059-021-02436-6)
Supplement: Supplementary file 1 — Additional file 1. Contains Supplementary Figures S1-S4. [file 13059_2021_2436_MOESM1_ESM.pdf]

Fig. S1

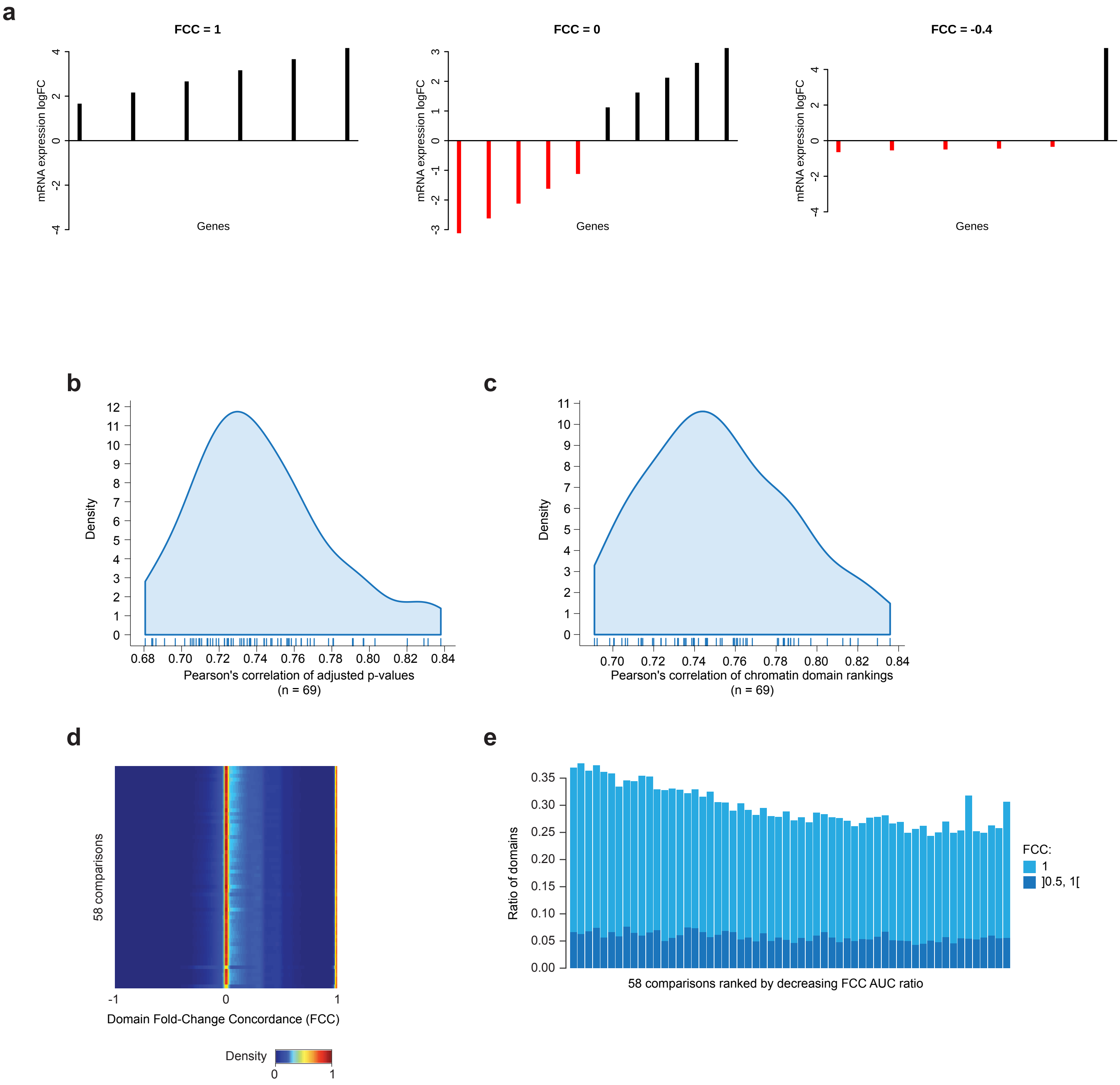
a
FCC = 1
FCC = 0
FCC = -0.4
mRNA expression logFC
Genes
b
Density
Pearson's correlation of adjusted p-values
(n = 69)
c
Density
Pearson's correlation of chromatin domain rankings
(n = 69)
d
58 comparisons
Domain Fold-Change Concordance (FCC)
Density
e
Ratio of domains
58 comparisons ranked by decreasing FCC AUC ratio
FCC:
1
]0.5, 1[

Fig. S1: Gene co-regulation in chromatin domains

a) Toy examples of domain fold-changes and associated FCC scores.

b-c) Distribution of the Pearson's correlation coefficients between domain adjusted p-values **(b)** and ranks **(c)** obtained with DADo for same pairs of conditions but using different Hi-C datasets.

d) Heatmap representation of the density distribution of FCC scores (range: -1 to 1; X-axis) for each of the 58 comparisons (Y-axis) after randomizing gene-to-domain assignments.

e) Ratio of concordant domains (0.5 < FCC < 1) and fully concordant domains (FCC = 1) for all comparisons (X-axis) ranked by decreasing FCC AUC ratio.

Fig. S2

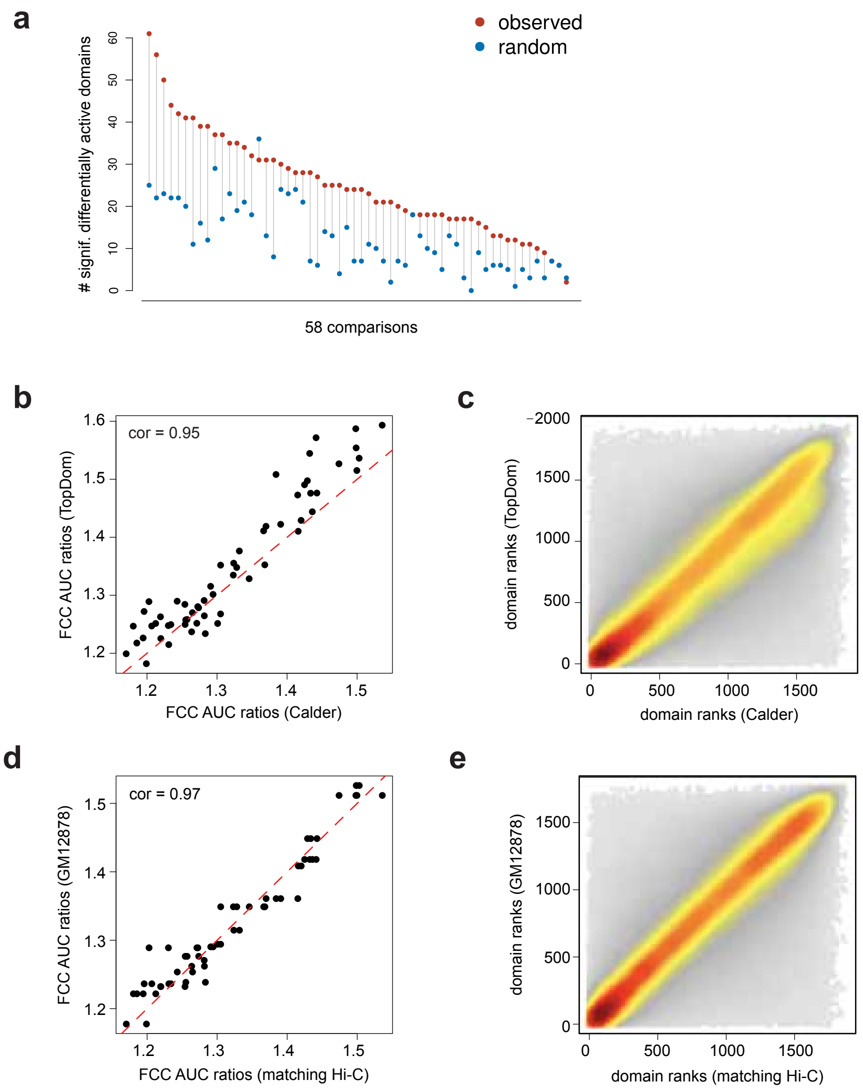
a
observed
random
signif. differentially active domains
0
10
20
30
40
50
60
58 comparisons
b
cor = 0.95
FCC AUC ratios (TopDom)
FCC AUC ratios (Calder)
c
domain ranks (TopDom)
domain ranks (Calder)
d
cor = 0.97
FCC AUC ratios (GM12878)
FCC AUC ratios (matching Hi-C)
e
domain ranks (GM12878)
domain ranks (matching Hi-C)

Fig. S2: Differentially active domains

a) For each comparison (X-axis), number of significant differentially active domains (Y-axis) detected by DADo for the observed (red) and random (blue) list of chromatin domains.

b-c) Comparison of FCC AUC ratios **(b)** and domain ranks **(c)** obtained for each comparison using compartment domains inferred by Calder (X-axis) or TADs inferred by TopDom (Y-axis).

d-e) Comparison of FCC AUC ratios **(d)** and domain ranks **(e)** obtained for each comparison using compartment domains inferred by Calder (X-axis) on each Hi-C dataset or always using the same domains inferred from the highest resolution Hi-C dataset (GM12878). The red line indicates the y=x line. The scatter plots comparing domain ranks are color coded by density of points (gray = low density, yellow-to-red = high density).

Fig. S3

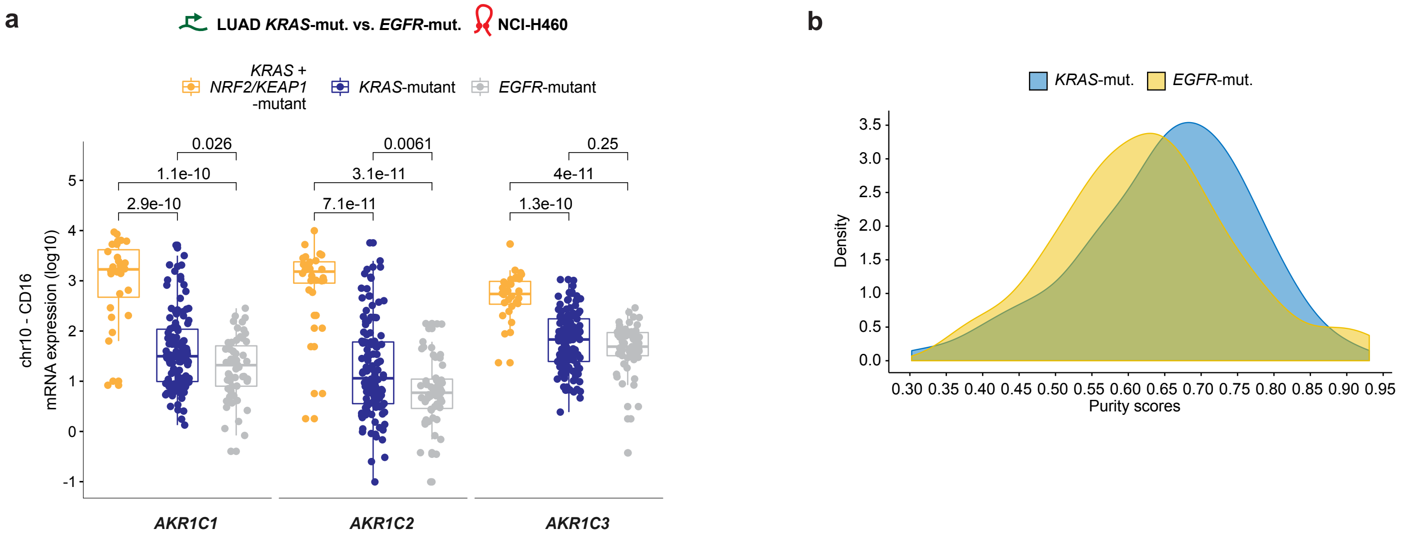
a
LUAD KRAS-mut. vs. EGFR-mut.
NCI-H460
KRAS + NRF2/KEAP1 -mutant
KRAS-mutant
EGFR-mutant
chr10 - CD16 mRNA expression (log10)
0.026
1.1e-10
2.9e-10
0.0061
3.1e-11
7.1e-11
0.25
4e-11
1.3e-10
AKR1C1
AKR1C2
AKR1C3
b
KRAS-mut.
EGFR-mut.
Density
Purity scores

Fig. S3: Differentially active domains in lung adenocarcinoma

a) Boxplot comparison of mRNA expression values (log10) for the genes belonging to chr10-CD16. *KRAS*-mutant samples are divided in those exhibiting either *NFE2L2* (NRF2) or *KEAP1* mutations (yellow) or not (blue). *EGFR*-mutant samples are in gray. Wilcoxon's test p-values are indicated.

b) Distribution of the tumor purity scores (X-axis) for the *KRAS*-mutant (blue) and *EGFR*-mutant (yellow) samples in lung adenocarcinoma.

Fig. S4

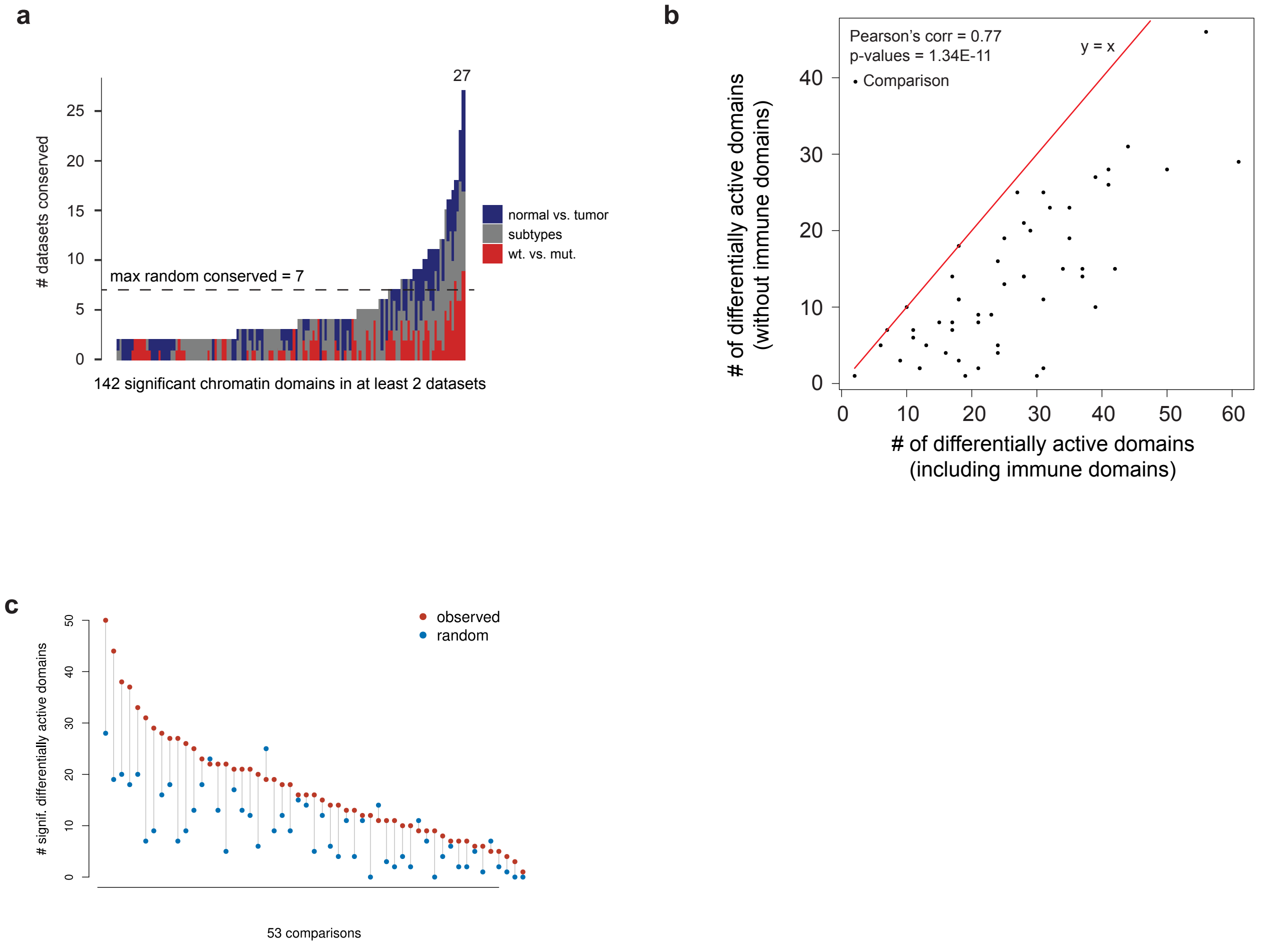
a
27
datasets conserved
max random conserved = 7
normal vs. tumor
subtypes
wt. vs. mut.
142 significant chromatin domains in at least 2 datasets
b
Pearson's corr = 0.77
p-values = 1.34E-11
• Comparison
y = x
# of differentially active domains
(without immune domains)
# of differentially active domains
(including immune domains)
c
observed
random
signif. differentially active domains
53 comparisons

Fig. S4: Conserved region and immune domains

a) Differentially active domains in more than one comparison (X-axis) are ranked by increasing numbers of datasets where they were found significant. Color coded are the categories of comparison and the dashed line (y = 7) indicates the maximal number of datasets in which random domains were found significant.

b) Number of domains detected as differentially active when running DADo on the initial datasets or after removing immune domains (Y-axis). Solid red line indicates the y=x curve.

c) For each comparison (X-axis), number of significant differentially active domains (Y-axis) detected by DADo for the observed (red) and random (blue) list of chromatin domains, after discarding immune domains.
